# Efficacy of *Boswellia serrata* Extract and/or an Omega-3-Based Product for Improving Pain and Function in People Older Than 40 Years with Persistent Knee Pain: A Randomized Double-Blind Controlled Clinical Trial

**DOI:** 10.3390/nu15173848

**Published:** 2023-09-03

**Authors:** Silvia Pérez-Piñero, Juan Carlos Muñoz-Carrillo, Desirée Victoria-Montesinos, Ana María García-Muñoz, Luis Andreu-Caravaca, Mario Gómez, Melanie Schölzel, Ana I. García-Guillén, Francisco Javier López-Román

**Affiliations:** 1 Faculty of Medicine, UCAM Universidad Católica San Antonio de Murcia, Carretera de Guadalupe s/n, 30107 Murcia, Spain; sperez2@ucam.edu (S.P.-P.); landreu@ucam.edu (L.A.-C.); igarcia53@ucam.edu (A.I.G.-G.); jlroman@ucam.edu (F.J.L.-R.); 2 Faculty of Pharmacy and Nutrition, UCAM Universidad Católica San Antonio de Murcia, 30107 Murcia, Spain; dvictoria@ucam.edu (D.V.-M.); amgarcia13@ucam.edu (A.M.G.-M.); 3 Faculty of Sports, UCAM Universidad Católica San Antonio de Murcia, 30107 Murcia, Spain; 4 Evonik Operations GmbH, Kirschenallee 45, 64293 Darmstadt, Germany; mario.gomez@evonik.com (M.G.); melanie.schoelzel@evonik.com (M.S.); 5 Primary Care Research Group, Biomedical Research Institute of Murcia (IMIB-Arrixaca), 30120 Murcia, Spain

**Keywords:** knee pain, omega-3, *Boswellia serrata*, docosahexaenoic acid, eicosapentaenoic acid, quality of life, sleep

## Abstract

A single-center, randomized, double-blind, controlled clinical trial with four arms was conducted in healthy subjects with persistent knee discomfort (pain intensity on 1–10 cm visual analog scale (VAS) > 3) aged 40 years and older treated with a dietary supplement for 8 weeks. The study groups were *Boswellia serrata* extract (*n* = 29), an omega-3-based product (AvailOm^®^ 50 High EPA) (*n* = 31), *Boswellia* + AvailOm^®^ (*n* = 30), and placebo (*n* = 30). The intake of *Boswellia* + AvailOm^®^ improved the quality of life (QoL) (WOMAC index) and some variables of muscle strength. Statistically significant differences between the AvailOm^®^ and the placebo groups in the decrease of pain intensity were found. Weekly VAS scores showed a significant decrease in pain perception when comparing the AvailOm^®^ product to the placebo, with the lowest VAS scores at week 8. Consumption of *Boswellia* improved sleep latency. The time to perform the Up and Go test decreased after the intake of AvailOm^®^. There was an increase in the omega-3 fatty acids, with the greatest increase in the *Boswellia* + AvailOm^®^ group. AvailOm^®^ was safe and effective in reducing pain and improving the QoL and functionality of subjects over 40 years with persistent knee pain.

## 1. Introduction

The ongoing demographic change and concomitant population aging as well as unhealthy lifestyles cause a rise in the prevalence of people experiencing joint discomfort [1].

Key prevention strategies include the reduction of the overuse of joints (e.g., related to workload or excessive sporting activities) and the promotion of healthy habits (e.g., regular physical exercise, adequate nutrition, maintaining a normal body weight).

Supportive preventative measures such as nutritional supplements are on the rise, but proper scientific evidence for specific ingredients is often rare.

If acute symptoms are not counteracted in time, in the worst case chronic conditions such as osteoarthritis can develop. Osteoarthritis (OA) is one of the main causes of functional disability and chronic pain. OA is a very prevalent condition, with a global increase of 113.25% from 1990 to 2019 [2], and the rising trend is likely to continue due to the aging of the population, the obesity epidemic, and the sedentary lifestyle [3,4]. Insufficient self-repair by the damaged joints associated with mechanical stress is considered to be involved in the development of OA [5]. OA is a leading condition of chronic pain, decreases the quality of life (QoL), increases work disability, and is a source of societal costs and economic burden for healthcare systems [6].

Knee OA is the most common subtype of OA. In 2020, it was estimated that there were around 654.1 million subjects (40 years or older) with knee OA worldwide, with a pooled global incidence of 203 per 10,000 person-years in persons of 20 years of age or more [7]. Major pathological findings in patients with OA include cartilage degeneration with subchondral bone alterations and synovitis [8]. Moreover, the progression of the disease is aggravated by aging, mechanical overload, metabolic factors, inflammation, and hormonal changes.

Patients with OA generally seek medical care because of pain, which is the cardinal symptom of the disease. The use of joints increases pain, whereas pain is relieved by resting. Stiffness and pain are typically intermittent at the beginning of OA, becoming more severe and frequent along the progression of the disease. However, the severity of the disease is based on changes shown on plain X-rays and the intensity of pain is poorly correlated [9], probably because radio-imaging studies are insensitive indicators of the structural and nociceptive pathway changes that occur in OA [10]. The main purpose of management in OA is to reduce pain and disability as well as to improve functionality and daily QoL by increasing physical activity and muscle strength [11]. The relief of pain in subjects with OA often requires a mix of therapeutic approaches, such as pharmacological treatment with different analgesic medication groups including non-steroidal anti-inflammatory drugs (NSAIDs) and opioid analgesics, knee arthroplasty when indicated in patients with disabling pain and progressive deformity and/or instability, and non-pharmacological treatment, particularly exercise and physical therapy [12]. However, the safety profiles of NSAIDs and opioids need to be considered and are not adequate treatment modalities for the long-term relief of pain [13].

Based on the limitations of efficacy and long-term safety of available pharmacological treatments, there has been an increasing interest in the identification of natural products with anti-oxidative and anti-inflammatory properties that may safely promote joint health and prevent OA. Omega-3 polyunsaturated fatty acids (PUFAs) especially eicosapentaenoic acid (EPA) and docosahexaenoic acid (DHA) commonly consumed in fatty fish and seafood, cereal products, seeds, nuts, and vegetables are recognized for their anti-inflammatory and pleiotropic properties associated with the formation of eicosanoids, resolvins, key proteins, cytokines, and mediators of inflammation, which play a role in reducing the low-grade inflammatory environment and cartilage degradation associated with OA [14,15,16,17,18]. Omega-3 products are popular dietary supplements due to their potential health benefits of reducing inflammation and oxidative stress damage and improving well-being. In the Australian Longitudinal Study on Women’s Health (ALSWH) based on a large nationally representative sample of 10,638 Australian women, the consumption of omega-3 was significantly more likely in the subgroup of women with joint pain [19].

On the other hand, pentacyclic triterpenic acids are the active ingredients in *Boswellia serrata* (*Salai*/*Salai guggul*) (Family: *Burseraceae*; Genus: *Boswellia*) and are responsible for the inhibition of pro-inflammatory cytokines, with 3-acetyl-11-keto-β-boswellic acid (AKBA) having a powerful inhibitory effect on 5-lipoxygenase [20,21]. Data collected from clinical studies showed that extracts of *Boswellia serrata* improved physical function and pain due to their anti-arthritis and anti-inflammatory properties [22,23]. In a recent systematic review and meta-analysis of seven randomized controlled trials involving 545 patients with OA, *Boswellia* or its extracts were significantly more effective than placebo, ibuprofen, or glucosamine sulfate (control group) in relieving pain and stiffness and improving joint function, with a recommended duration of treatment of at least 4 weeks [24].

Therefore, it was considered of interest to design a clinical trial to assess the efficacy of a dietary supplement of an omega-3-based product given alone or combined with a *Boswellia serrata* extract as compared with the *Boswellia serrata* extract or placebo to relieve pain and improve joint functionality in healthy people of more than 40 years of age with knee pain.

## 2. Materials and Methods

### 2.1. Design and Participants

This was a single-center, randomized, double-blind, and controlled study with four arms carried out at the Health Sciences Department of Universidad Católica San Antonio de Murcia (UCAM), in Murcia, Spain. The study period was from 26 January to 18 November 2022. The primary objective of the study was to assess the efficacy of a combination product containing omega-3 fatty acids as well as *Boswellia serrata* extract consumed for 60 days on the intensity of pain in healthy people older than 40 years suffering from persistent knee pain. Secondary objectives included changes in QoL, functional mobility, muscle strength, sleep quality, level of physical activity, body composition, omega-3 fatty acids bioavailability, and safety.

Participants were mainly recruited by advertising the study through mass media and social networks available at UCAM University. Eligibility included subjects of both sexes in an age range of 40 to 75 years with persistent unilateral knee pain (> 3 using a 1–10 cm visual analog scale (VAS)). When knee pain was bilateral, the knee with the highest VAS score was chosen. Exclusion criteria were as follows: previous use of OA medications such as NSAIDs, opiates, or immunosuppressants; current treatment with chondroitin sulfate, glucosamine, or hyaluronic acid intra-articular injections; use of any supplement for improving health joints; chronic inflammatory diseases of the musculoskeletal system (such as gout, rheumatoid arthritis, Paget’s disease, chronic pain syndrome, etc.); severe or terminal illness; obesity (body mass index (BMI) > 32 kg/m^2^); current use (or use in the previous 2 months) of omega-3-based supplements and/or supplements based on the botanical ingredient under investigation; known allergy to any of the study components; breast-feeding or pregnant women; and inability to provide informed consent.

The study protocol was approved by the Ethics Committee of Universidad Católica San Antonio de Murcia (code CE062105; approval date 25 June 2021) (Murcia, Spain) and was registered in the ClinicalTrials.gov (NCT05279573). All participants provided written informed consent.

### 2.2. Intervention and Study Procedures

Participants were randomly assigned in equal proportion to one of the four study groups using a simple randomization procedure with the Epidat 4.1 software program. The study groups were as follows: (a) *Boswellia serrata* extract, (b) the omega-3-based active product, (c) *Boswellia serrata* extract plus the omega-3-based active product; and (d) placebo.

The tablets of the products under investigation were *Boswellia serrata* extract 12.5% film-coated tablets, AvailOm^®^ 50 High EPA (“AvailOm^®^”, consisting of lysine salts of EPA and DHA) 25% + lecithin 12.9% film-coated tablets, *Boswellia serrata* extract 12.5% + AvailOm^®^ 25% + lecithin 12.9% film-coated tablets, and placebo. All investigational products were provided by Evonik Operations GmbH, Darmstadt, Germany. All participants were instructed to take 4 tablets a day (2 tablets at the time of breakfast and 2 tablets at the time of dinner) for 8 consecutive weeks (60 days). Dose compliance in percentage was defined as the number of tablets taken by the participant during the study divided by the number of tablets expected to be taken (*n* = 240) and multiplied by 100. Subjects were required to consume at least 80% of the tablets so that only 48 tablets could be left corresponding to 12 days out of 60 days of consumption. It was strongly recommended not to make changes to the dietary habits. The use of any new medication should be reported to the principal investigator, and any analgesic or other medications taken during the course of the study should be recorded in the diary card.

Participants visited the institute within ± 7 days of the baseline visit, in which the written informed consent was obtained, the inclusion criteria were checked, and randomization was performed. The study included a baseline visit (visit 1) and a visit after 60 days (visit 2, end of study). Participants also received a telephone call on day 30 to check adherence with the assigned product. Intensity of pain, QoL, functional mobility, muscle function, sleep quality, level of physical activity, and body composition were assessed at visits 1 and 2. A blood sample was drawn at visits 1 and 2 for routine safety testing. At visit 2, adverse events were recorded, and the study product was collected.

### 2.3. Study Variables

Clinical variables included age, sex, body mass index (BMI), and blood pressure. Pain was the primary efficacy variable and was measured using a 1–10 cm VAS scale (0 = no pain, 10 = worst imaginable pain), with scores < 4 categorized as mild pain, scores between 4 and 6 as moderate pain, and > 6 as severe pain. The intensity of pain was evaluated at visits 1 and 2 before muscle strength testing and at weekly intervals as self-reported by participants. The VAS scores were assessed each morning upon waking, with reference to the pain intensity encountered on the preceding day. Every day, the participants’ VAS scores were documented in individualized notebooks.

Health-related QoL was evaluated according to the impact of the three subscales of pain (5 items), stiffness (2 items), and physical function (17 items) of the Western Ontario McMaster Universities Arthritis Index (WOMAC). Subjects were asked to answer each question with regard to pain, stiffness, or difficulty experienced in the previous 48 h. The test questions were scored on a scale of 0–4 (0 = none, 1 = slight, 2 = moderate, 3 = severe, 4 = extreme). Higher scores on the WOMAC indicate worse pain, stiffness, and functional limitations. A Spanish-validated version of the WOMAC index was administered [24].

The Timed Up and Go test (TUG) was used to assess balance and mobility. Briefly, participants were seated correctly in a chair without using the armrests. They were instructed to stand up from the chair, walk a distance of 3 m, turn back to the chair, and then sit down. The time taken for this task, measured with a stopwatch, represented the individual’s TUG score. Two trials were performed with an interval of 30 s, and the average time was taken as the final score. A TUG score of 13.5 s or higher indicated a higher risk of falling, while a score below 13.5 s suggested better functional performance [25].

Isokinetic and isometric dynamometry were used for the measurement of the muscle function of the knee. The Biodex System 3 (Biodex Medical Systems, Shirley, NY, USA) isokinetic dynamometer was used for isokinetic dynamometry tests. Exercises were performed on a cycle ergometer (W45, 70–75 rpm) with the alignment of the dynamometer and knee movement axes, and the resistance pad was fixed on the distal tibia (two-thirds). A warming-up period of 5 min was required. The initial value was taken as the active maximal knee extension (0–90° motion range). Then, five repetitions of maximal flexion and extension of the knee at 60° and 180°·s^−1^ were performed, with two previous sets of repetitions as warm-up exercises. The following variables were measured: peak torque isokinetic value (Newton meters (Nm)), total work (TW) (Joules (J)), average power measured (watts (W)), and total word for 1 RM (repetition maximum) (J). In the isometric dynamometry testing at 90°·s^−1^, the force trying to completely extend the knee, with the lever not allowing to perform any movement was measured. After one warm-up test, subjects were instructed to perform three repetitions, with the force maintained for 5 s and a resting interval of 30 s. Variables recorded included peak isometric torque (Nm) and average peak isometric torque (Nm).

Sleep quality was evaluated by actigraphy (ActiGraph wGT3X-BT accelerometer, ActiGraph, Pensacola, FL, USA), and the following variables were recorded: sleep latency, sleep efficiency, total time in bed, total sleep time, wakefulness after sleep onset, number of awakenings, and the average number in minutes of awakenings. The Pittsburgh Sleep Quality Index (PSQI) was also administered. It is a self-rated questionnaire that assesses sleep quality over an interval of 1 month. The overall score ranges between 0 and 21 and is the sum of seven components (sleep latency, subjective sleep quality, duration of sleep and sleep disturbances, habitual sleep efficiency, need for medication to sleep, and daytime dysfunction). Lower scores indicate better sleep quality. A Spanish-validated version of the PSQI was used [26].

Capillary puncture in dried blood sample spots was the technique used for measurement of EPA and DHA bioavailability, with results expressed as % of fatty acids. We also calculated the EPA + DHA omega-3 index. A triaxial accelerometer was used to assess the physical activity level, with results expressed as metabolic equivalents (METs). A whole-body bioimpedance analyzer (Tanita BC-420MA, Tanita Corp., Tokyo, Japan) was used to determine corporal composition (body mass index (BMI), percentage of fat mass). Safety data included blood pressure recording and laboratory analyses. Standard hematological (hemogram) and biochemical parameters (renal and liver function tests) were measured.

### 2.4. Statistical Analysis

The sample size was calculated according to the intensity of joint pain (VAS) as the primary efficacy endpoint. On the basis of a standard deviation of VAS score of 1.48 reported in a similar population [27], for a precision of 1 with an alpha risk of 5% and statistical power of 80%, 28 subjects were needed in each group, which was increased to 31 subjects per group according to a 10% loss to follow-up.

Data of all participants who fulfilled the inclusion criteria and completed the 8-week period of the trial were analyzed. Frequencies and percentages are used for the expression of categorical variables, and mean ± standard deviation (SD) is used for the expression of continuous variables. The chi-square test or Fisher’s exact test was used for the comparison of categorical variables between the study groups, and Student’s *t*-test was used for the comparison of quantitative variables. The analysis of variance (ANOVA) for repeated measures was used to assess the change of variables corresponding to each group throughout the study period. The subject factor included data at baseline and at 8 weeks, and the between-subject factor for paired data included the product administered, that is *Boswellia*, AvailOm^®^ or *Boswellia* + AvailOm^®^, and placebo. Turkey’s or Bonferroni’s correction was applied for post-hoc analyses. A *p* < 0.05 was considered statistically significant. Data analyses were performed with the SPSS version 25.0 (IBM Corp., Armonk, NY, USA) software program.

## 3. Results

### 3.1. Participants

**Figure 1:**
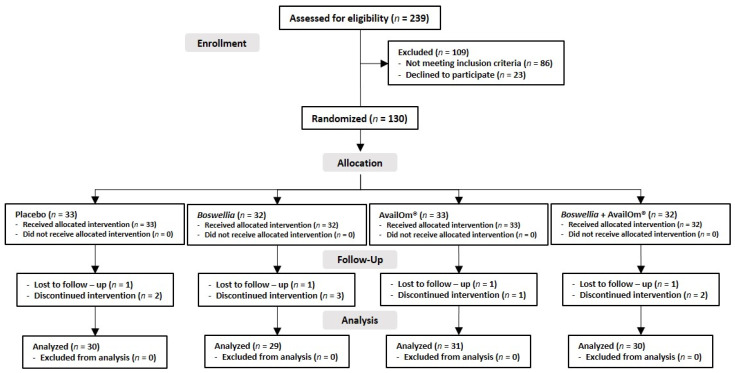
Distribution of the participants in the four study groups.

A total of 239 subjects were assessed for eligibility and 130 were randomized into the four study groups, but 10 subjects did not complete the follow-up. Finally, 120 subjects (*Boswellia*, *n* = 29; AvailOm^®^, *n* = 31; *Boswellia* + AvailOm^®^, *n* = 30; placebo, *n* = 30) were included (Figure 1).

The study population included 59 men and 61 women, with a mean age of 51.2 ± 8.3 years, without statistically significant differences in baseline data (Table 1).

### 3.2. Quality of Life Assessed by the WOMAC Index

Results of scores of the WOMAC index in the overall assessment of QoL and in the items of pain, stiffness, and physical function are shown in Table 2.

**Figure 2:**
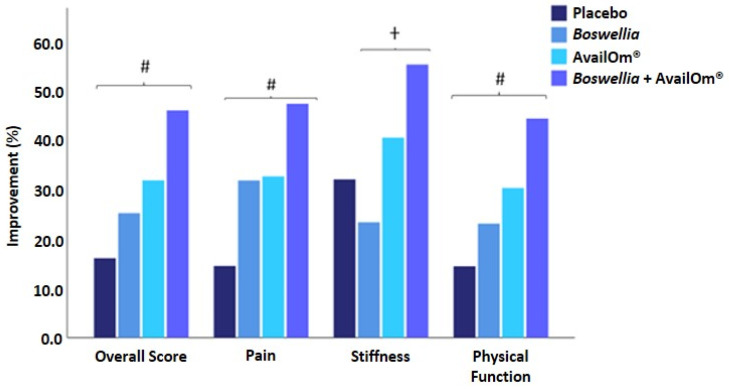
Improvements (%) in the Western Ontario McMaster Universities Arthritis Index parameters (# *p* < 0.05 *Boswellia* + AvailOm^®^ vs. placebo; † *p* < 0.05 *Boswellia* vs. *Boswellia* + AvailOm^®^).

The overall WOMAC score decreased in all groups at the end of the study as compared with baseline, with statistically significant between-group and within-group differences. However, mean differences of decreases of WOMAC overall score were only statistically significant for the group of *Boswellia* + AvailOm^®^ as compared with placebo (mean −8.327 ± 2.788, *p* = 0.021). In the items of pain, decreases in WOMAC scores at the end of the study were significant for all active supplementation groups (*p* = 0.001), whereas in the placebo group, statistical significance was not reached (*p* = 0.064). Between-group differences were significant (*p* = 0.05), but in the pairwise comparisons of groups, mean differences were only significant for *Boswellia* + AvailOm^®^ vs. placebo (mean: −2.086 ± 0.631, *p* = 0.008). In the items of stiffness, all scores decreased significantly at the end of the study, except for the *Boswellia* group (*p* = 0.129). Between-group differences were statistically significant (*p* = 0.029), although, in the pairwise comparisons, the decrease of stiffness in the *Boswellia* + AvailOm^®^ group was greater than in the *Boswellia* group (mean difference: 1.131 ± 0.427, *p* < 0.05). In relation to physical function, there were statistically significant decreases in WOMAC scores in all groups except for the placebo group (*p* = 0.101). Between-group differences were statistically significant (*p* = 0.013), and in the pairwise comparisons, *Boswellia* + AvailOm^®^ showed a better functional improvement than placebo (mean difference: −5.616 ± 2.098, *p* < 0.05). Figure 2 compares each of the products for each of the variables evaluated with the WOMAC questionnaire.

### 3.3. Pain Intensity

#### 3.3.1. Initial and Final VAS Scores

In all study groups, VAS scores of pain intensity showed a statistically significant decrease at the end of the study as compared with baseline (Table 3). Also, within-group analyses showed statistically significant differences in VAS scores of the three groups of *Boswellia*, AvailOm^®^, and *Boswellia* + AvailOm^®^ as compared to placebo, with mean differences of −1.784 ± 0.499 (*p* = 0.003), −1.779 ± 0.490 (*p* = 0.003), and −1.695 ± 0.495 (*p* < 0.005), respectively.

#### 3.3.2. Weekly VAS Scores

As shown in Table 4, weekly VAS scores of pain intensity decreased significantly in all study groups from baseline to the end of the study, with statistically significant within-group and between-group differences. The mean difference in the pairwise comparison of groups was only statistically significant for the AvailOm^®^ group vs. placebo (mean: −1.465 ± 0.538, *p* = 0.045).

### 3.4. Time up and Go (TUG) Test

TUG decreased significantly in the *Boswellia* + AvailOm^®^ group from a mean of 6.16 ± 0.87 s to 5.68 ± 0.72 at the end of the study (*p* = 0.001), as well as in the AvailOm^®^ group, which went from a mean of 6.08 ± 1.22 s at baseline to 5.59 ± 0.90 s at the end of the study (*p* = 0.001). Significant differences in the other groups of *Boswellia* or placebo were not observed. Between-group differences were significant (*p* < 0.05), and in the pairwise comparisons, AvailOm^®^ was superior to *Boswellia* (mean difference: −0.391 ± 0.157, *p* < 0.05).

### 3.5. Muscle Strength: Isokinetic and Isometric Dynamometry

The details of results obtained in isokinetic and isodynamic dynamometry variables are shown in Table 5. In the exercises of isokinetic dynamometry testing, improvements in knee function limitation were observed in the groups of subjects assigned to supplementation with the combination of *Boswellia* + AvailOm^®^ and, to some extent, to the group supplemented with AvailOm^®^. At 60° position with knee extension, statistically significant improvements in peak torque, total work for maximum repetition, and average power were found. Also, at 60° with knee flexion, subjects in the *Boswellia* + AvailOm^®^ group improved significantly in all parameters (peak torque, total work, total work for maximum repetition, and average power). At 180° with knee flexion and extension, subjects treated with AvailOm^®^ or *Boswellia* + AvailOm^®^ improved in peak torque, total work, total work for maximum repetition, and average power, with statistically significant differences at the end of the study in comparison with baseline.

In isometric dynamometry testing, significant improvements in peak torque and average peak torque were only observed in the *Boswellia* + AvailOm^®^ group (Table 5).

### 3.6. Sleep Quality

Results of the PSQI showed statistically significant improvements in the quality of sleep in the AvailOm^®^ group, with a mean value of 7.4 ± 3.8 at baseline and 6.4 ± 3.1 at the end of the study (*p* = 0.023).

**Figure 3:**
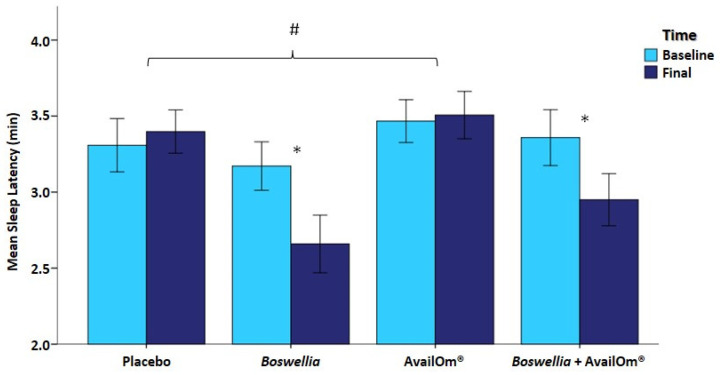
Differences in sleep latency with significant between-group differences in the *Boswellia* (* *p* = 0.016) and *Boswellia* + AvailOm^®^ (* *p* = 0.049) groups. Mean differences were significant (# *p* < 0.05) between *Boswellia* vs. placebo and *Boswellia* vs. AvailOm^®^.

Actigraphy sleep studies showed significant decreases in sleep latency in the *Boswellia* and *Boswellia* + AvailOm^®^ groups at the end of the study as compared with baseline. Mean differences were significant for *Boswellia* vs. placebo (−0.708 ± 0.231, *p* = 0.016) and *Boswellia* vs. AvailOm^®^ (−0.781 ± 0.230, *p* = 0.006) (Figure 3).

Sleep efficiency improved significantly in the *Boswellia* group only. Total time in bed, total sleep time, and average minutes in awakenings did not show significant changes in any of the study groups. However, wakefulness after sleep onset and number of awakenings showed a significant decrease in *Boswellia* as well as *Boswellia* + AvailOm^®^ groups at the end of the study in comparison with baseline. Table 6 shows the details of the results obtained in the sleep assessment by actigraphy.

### 3.7. Bioavailability of Fatty Acids

In relation to the omega-3 index, there were statistically significant differences in the within-group comparisons except for the placebo group. Between-group differences were statistically significant (*p* = 0.001) (Table 7). In the pairwise comparisons, the groups of AvailOm^®^ and *Boswellia* + AvailOm^®^ showed significant differences with the placebo group (mean differences 0.683 ± 0.211, *p* = 0.009 and 0.753 ± 0.214, *p* = 0.004, respectively) and the *Boswellia* group (mean differences 0.786 ± 0.214, *p* = 0.002 and 0.856 ± 0.218, *p* = 0.001, respectively).

Changes in DHA showed statistically significant increases in the *Boswellia* and *Boswellia* + AvailOm^®^ groups at the end of the study, whereas EPA increased significantly in the AvailOm^®^ and *Boswellia* + AvailOm^®^ groups as compared with baseline (Table 7). In addition, AvailOm^®^ and Bos*wellia* + AvailOm^®^ groups showed significant differences in EPA values with the placebo group (mean differences: 0.422 ± 0.080, *p* < 0.0001, and 0.424 ± 0.080, *p* < 0.001, respectively) and the *Boswellia* group (mean differences: 0.490 ± 0.080, *p* < 0.0001, and 0.492 ± 0.081, *p* < 0.001, respectively).

### 3.8. Level of Physical Activity

Changes in the level of physical activity were not significant in any study group. At baseline and at the end of study, the mean values were 1.6 ± 0.2 and 1.6 ± 0.2 METs in the placebo group (*p* = 0.904), 1.5 ± 0.2 and 15.02 METs in the *Boswellia* group (*p* = 0.858), 1.6 ± 0.2 and 1.6 ± 0.2 METs in the AvailOm^®^ group (*p* = 0.946), and 1.6 ± 0.2 and 1.6 ± 0.2 METs in the *Boswellia* + AvailOm^®^ group (*p* = 0.975).

### 3.9. Anthropometric Variables, Blood Pressure, and Safety

Changes in BMI, percentage of fat mass, and SBP and DBP during the study period were not observed. Also, the results of laboratory tests remained within the normal ranges. Adverse events of mild intensity and unrelated to the study product were recorded in 21 cases, which were mostly gastrointestinal complaints, including constipation, diarrhea, and heartburn.

## 4. Discussion

In this randomized double-blind and controlled clinical study, dietary supplementation with an omega-3-based product administered for 8 weeks was effective in reducing persistent knee pain or discomfort in subjects over 40 years of age. Health-related QoL is receiving increasing attention as an outcome measure for knee pain based on scores of generic and knee-specific questionnaires, such as the WOMAC index. The self-administered WOMAC index is the most commonly used clinical tool for evaluating patients with knee OA [28]. In the present study, the use of AvailOm^®^ alone or combined with *Boswellia* was associated with a significant improvement in QoL, with statistically significant decreases in the overall WOMAC scores as well in the items of pain, stiffness, and physical function. In a meta-analysis of the impact of nutritional supplementation on OA symptoms, improvement in WOMAC function was reported after omega-3 supplementation for 12 or 24 weeks [29].

The benefits of dietary supplementation extend beyond just improvements in WOMAC scores. In this study, other important findings were a decrease in the time required to perform the TUG test after consuming AvailOm^®^, as well as improvement in muscle strength in some variables of isokinetic and isometric dynamometry studies in the groups of AvailOm^®^ alone and combined with *Boswellia*. These findings align with benefits in TUG, muscle mass gain, and improved walking speed reported in a meta-analysis of 10 studies of omega-3 supplementation in elderly people [30]. In healthy older adults, omega-3 supplementation improved isometric strength, which has been attributed to changes in muscle quality and the capacity to stimulate muscle anabolism [31]. Moreover, data from a systematic review and meta-analysis of 16 articles involving omega-3 fatty acid supplementation on measures of muscle mass and function in older adults confirmed the benefits of improving lower body strength, TUG, and sit-to-stand performance repetitions [32].

Furthermore, our study showed a significant decrease in pain perception when comparing the AvailOm^®^ product to the placebo, with the lowest VAS scores being recorded during the last week of the study (week 8). From week 6 to week 8, subjects assigned to the AvailOm^®^ group showed the lowest VAS scores as compared not only with placebo but also with the *Boswellia*-containing groups. The weekly VAS scores for this combination group showed a significant decrease in pain perception from baseline to the end of the study, suggesting a potential synergistic effect of *Boswellia* and AvailOm^®^ on pain reduction.

The efficacy of these dietary supplements aligns with a larger body of literature supporting the analgesic effects of foods and nutrients. Omega-3 fatty acid supplements, in particular, have been shown to reduce OA pain due to their anti-inflammatory and anti-nociceptive actions [33]. For instance, a recent study of the Seniors–ENRICA-1 cohort in Spain, which included 950 individuals aged ≥ 60 years, reported that higher oily fish consumption was associated with reduced pain incidence and worsening over 5 years; higher marine omega-3 fatty acid intake (including EPA and DHA) was linked to less pain worsening [34]. Furthermore, a meta-analysis reported a significant reduction in patient-reported joint pain intensity, minutes of morning stiffness, number of painful and/or tender joints, and NSAID consumption following 3–4 months of omega-3 PUFA supplementation [35].

Other randomized double-blind placebo-controlled studies have shown the analgesic efficacy and safety of *Boswellia serrata* extract in patients with OA of the knee [22,23]. The mechanism of the anti-inflammatory activity of *Boswellia* extracts is due to some boswellic acids, particularly AKBA [36]. Specialized pro-resolving mediators (SPM) play a crucial role in resolving inflammation, with 15-lypoxygenase-1 (15-LOX-1) as a key factor for SPM biosynthesis. It has been shown that AKBA activates cellular 15-LOX-1 via an allosteric site accomplishing robust SPM formation in M2 macrophages, promoting inflammation resolution [36]. In addition, the inflammation-resolving activities of DHA and EPA have been linked to the generation of SPM [37].

The results are quite conclusive in this regard. The sleep variables that were modified by the consumption of some products are latency, wakefulness, and awakenings (number). None of these variables were modified in the group of subjects that consumed AvailOm; on the other hand, the subjects that consumed Boswellia improved their score in these variables. In the group that consumed Boswellia and AvailOm, the change that occurred was very similar to the change in the group that consumed only Boswellia. Therefore, with these results, it does not appear that AvailOm can modify these variables even as an enhancing mechanism.

In relation to results regarding sleep variables, the consumption of *Boswellia* for 8 weeks has shown improvements in sleep latency, wakefulness after sleep onset, and number of awakenings. Any of these variables were modified in the group of subjects that consumed AvailOm^®^ only. In the group that consumed *Boswellia* and AvailOm^®^, the change that occurred was very similar to the change in the group that consumed only *Boswellia*. Therefore, with these results, it does not appear that AvailOm^®^ (at least at the doses used in the present study) can modify these variables even as an enhancing mechanism. In fact, data on the specific effect of *Boswellia* on sleep quality is limited. In four patients with chronic cluster headaches and disturbed sleep, oral *Boswellia serrata* extract reduced the intensity and frequency of the headaches and improved sleep [38]. However, there is evidence that pain and sleep can be interconnected [39], and it is known that OA pain, in particular nocturnal knee pain, increases the risk of sleep disturbances, which may trigger disability and depressive symptoms [40,41]. It seems that by reducing the underlying inflammation-related pathways of pain, benefits in better sleep can be obtained.

As may be expected, the consumption of the product with AvailOm^®^ produced an increase in the omega-3 index, DHA, and EPA, with the greatest increase in the *Boswellia* + AvailOm^®^ group. On the other hand, the intake of the products under investigation was safe and did not modify the physical activity levels or induce a change in body composition or alterations in laboratory parameters.

Limitations of the study include the reduced sample size and the duration of treatment of 8 weeks only. However, in relation to the selection of an 8-week supplementation period, in a previous publication of our group using omega-3 supplementation with DHA, activity and incorporation of the product into phospholipids of the erythrocyte membrane were observed at 30 days of starting supplementation with different doses [42]. Due to the dose used in the present study, it was preferred to prolong the duration of supplementation up to 2 months. In the case of *Boswellia*, there are different studies that have evaluated the efficacy of the product in the treatment of pain in knee OA with duration of supplementation of 60 days [22], and even recently, in 2023, satisfactory results at 30 days have been reported [43]. Subjective pain intensity assessed by VAS could be influenced by other factors, and the inclusion of imaging endpoints in future studies would provide further support to our findings. Also, strict control of dietary intake was not performed, although subjects were strongly advised against introducing changes in their dietary habits over the course of the study.

## 5. Conclusions

The combination of AvailOm^®^ with a *Boswellia extract* improved the QoL, TUG, some variables of isokinetic and isometric dynamometry, and sleep latency in subjects with persistent knee pain that were older than 40 years of age. Additionally, the consumption of AvailOm^®^ alone for 8 weeks was associated with a significant improvement in the subjective perception of knee pain compared to placebo. However, further randomized controlled studies are necessary to confirm these findings.

## Figures and Tables

**Table 1:** Demographic and clinical data at baseline.

Variables	Study Groups					
	Placebo (*n* = 30)	*Boswellia* (*n* = 29)	AvailOm^®^ (*n* = 31)	*Boswellia* + AvailOm^®^ (*n* = 30)	Total (*n* = 120)	*p* Value
Age, years	50.2 ± 8.1	50.5 ± 8.2	51.1 ± 6.8	53.2 ± 10.1	51.3 ± 8.4	0.510
Weight, kg	80.1 ± 15.6	76.1 ± 17.9	76.4 ± 14.7	76.4 ± 14.1	77.2 ± 15.5	0.717
Fat mass, %	28.5 ± 8.4	28.8 ± 7.4	29.0 ± 8.2	30.8 ± 8.4	29.3 ± 8.1	0.680
BMI, kg/m^2^	27.0 ± 3.5	25.8 ± 4.3	26.3 ± 3.7	26.9 ± 3.4	26.5 ± 3.7	0.562
Systolic BP, mmHg	124.4 ± 17.6	118.8 ± 20.6	120.9 ± 11.5	121.0 ± 15.1	121.3 ± 16.4	0.625
Diastolic BP, mmHg	79.6 ± 11.1	76.0 ± 12.7	78.0 ± 9.1	80.2 ± 11.1	78.5 ± 11.0	0.481
VAS score, cm	5.0 ± 1.7	5.5 ± 1.8	5.4 ±1.5	5.3 ± 1.9	5.3 ± 1.7	0.896

Data as mean ± standard deviation; BMI: body mass index; BP: blood pressure; VAS: visual analog scale.

**Table 2:** Changes in WOMAC scores in the four study groups.

WOMAC Score	Baseline	Final (8 Weeks)	% Improvement Due to Product	Within-Group *p* Value	Between-Group *p* Value
Overall					
Placebo	29.3 ± 13.5	24.6 ± 13.3	-	0.047	0.005
*Boswellia*	24.4 ± 14.0	18.2 ± 10.0	9.4	0.011	
AvailOm^®^	28.0 ± 10.7	19.1 ± 13.2	15.7	0.001	
*Boswellia* + AvailOm^®^	34.5 ± 12.8	18.7 ± 11.5	29.7	0.001	
Pain					
Placebo	6.4 ± 3.1	5.5 ± 2.9	-	0.064	0.005
*Boswellia*	5.9 ± 2.2	4.0 ± 2.3	18.1	0.001	
AvailOm^®^	5.9 ± 2.5	4.0 ± 2.9	18.1	0.001	
* Boswellia* + AvailOm^®^	7.4 ± 2.5	3.9 ± 2.8	33.2	0.001	
Stiffness					
Placebo	2.5 ± 1.3	1.7 ± 1.5	-	0.011	0.029
*Boswellia*	2.1 ± 1.7	1.6 ± 1.6	−8.0	0.129	
AvailOm^®^	2.9 ± 1.9	1.7 ± 1.5	9.4	0.001	
*Boswellia* + AvailOm^®^	3.2 ± 1.6	1.4 ± 1.4	24.3	0.001	
Physical function					
Placebo	20.3 ± 10.3	17.4 ± 10.3	-	0.101	0.013
* Boswellia*	16.4 ± 10.9	12.7 ± 7.3	8.4	0.038	
AvailOm^®^	19.2 ± 7.5	13.4 ± 9.7	16.0	0.001	
* Boswellia* + AvailOm^®^	23.9 ± 9.6	13.3 ± 8.5	30.2	0.001	

Data as mean ± standard deviation. WOMAC: Western Ontario McMaster Universities Arthritis index; % improvement due to product: percentage of improvement of each product after eliminating the placebo effect of the control group.

**Table 3:** VAS scores of pain intensity at baseline and at the end of the study.

Study Groups	VAS Score, Mean ± SD		% Improvement Due to Product	Within-Group *p* Value	Between-Group *p* Value
	Baseline	Final (8 Weeks)			
Placebo (*n* = 30)	5.1 ± 1.7	4.0 ± 2.2	-	0.006	0.001
*Boswellia* (*n* = 29)	5.4 ± 1.8	2.4 ± 1.8	29.6	0.001	
AvailOm^®^ (*n* = 31)	5.3 ± 1.5	2.3 ± 2.2	30.7	0.001	
*Boswellia* + AvailOm^®^ (*n* = 30)	5.4 ± 1.9	2.4 ± 2.0	29.6	0.001	

VAS: visual analog scale; SD: standard deviation, % improvement due to product: percentage of improvement of each product after eliminating the placebo effect of the control group.

**Table 4:** Weekly changes of VAS scores of pain intensity in the four study groups.

Time Point	Study Groups, VAS Score, Mean ± SD			
	Placebo (*n* = 30)	*Boswellia* (*n* = 29)	AvailOm^®^ (*n* = 31)	*Boswellia* + AvailOm^®^ (*n* = 30)
Baseline	5.0 ± 1.7	5.0 ± 1.8	5.4 ± 1.5	5.3 ± 1.9
Week 1	3.8 ± 2.0	3.6 ± 2.2	3.6 ± 2.2	3.7 ± 2.1
Week 2	3.6 ± 2.1	3.4 ± 2.1	3.3 ± 2.3	3.5 ± 1.9
Week 3	3.5 ± 2.0	3.2 ± 2.2	3.1 ± 2.3	3.2 ± 2.2
Week 4	3.4 ± 2.1	2.8 ± 1.9	2.7 ± 2.0	2.8 ± 2.2
Week 5	3.3 ± 2.0	2.4 ± 2.0	2.5 ± 2.0	2.8 ± 2.1
Week 6	3.3 ± 2.3	2.3 ± 2.0	2.2 ± 1.7	2.9 ± 2.5
Week 7	3.2 ± 2.2	2.3 ± 2.0	1.9 ± 1.6	2.6 ± 2.5
Week 8	3.2 ± 2.4	2.1 ± 1.9	1.8 ± 1.6	2.3 ± 2.3
Within-group *p* value	0.002	0.001	0.001	0.001
Between-group *p* value	0.010			

**Table 5:** Changes in muscle function of knee isokinetic and isodynamic dynamometry in the study groups.

		Study Group	Baseline	Final	Within-Group *p* Value	Between-Group *p* Value
**Isokinetic Dynamometry**						
At 60°·s^−1^ knee extension	Peak torque, Nm	Placebo	123.9 ± 59.5	120.0 ± 61.9	0.250	0.050
		*Boswellia*	104.3 ± 50.9	108.7 ± 45.7	0.205	
		AvailOm^®^	113.5 ± 50.6	121.4 ± 48.9	0.019	
		*Boswellia* + AvailOm^®^	109.5 ± 45.2	115.5 ± 48.5	0.050	
	Total work, J	Placebo	531.4 ± 241.4	524.8 ± 265.6	0.714	0.577
		*Boswellia*	464.2 ± 219.7	476.3 ± 192.3	0.508	
		AvailOm^®^	496.3 ± 214.7	524.9 ± 212.2	0.107	
		*Boswellia* + AvailOm^®^	472.9 ± 190.2	487.8 ± 213.6	0.409	
	Total work for 1RM, J	Placebo	114.0 ± 50.1	111.8 ± 54.3	0.494	0.235
		*Boswellia*	100.5 ± 46.4	103.2 ± 40.9	0.388	
		AvailOm^®^	106.1 ± 44.2	111.8 ± 43.1	0.050	
		*Boswellia* + AvailOm^®^	103.2 ± 39.4	108.9 ± 42.8	0.048	
	Average power, W	Placebo	73.7 ± 36.6	74.1 ± 41.4	0.847	0.050
		*Boswellia*	63.7 ± 31.1	66.1 ± 29.0	0.268	
		AvailOm^®^	68.5 ± 30.8	73.4 ± 29.7	0.021	
		*Boswellia* + AvailOm^®^	63.9 ± 28.0	71.8 ± 32.3	0.001	
At 60°·s^−1^ knee flexion	Peak torque, Nm	Placebo	62.0 ± 28.4	62.9 ± 30.6	0.690	0.223
		*Boswellia*	56.3 ± 31.2	58.3 ± 29.6	0.370	
		AvailOm^®^	61.1 ± 32.4	67.5 ± 32.8	0.003	
		*Boswellia* + AvailOm^®^	56.0 ± 26.0	61.0 ± 29.5	0.020	
	Total work, J	Placebo	313.6 ± 159.6	312.9 ± 165.7	0.957	0.131
		*Boswellia*	282.7 ± 185.8	292.0 ± 170.7	0.460	
		AvailOm^®^	313.8 ± 183.7	343.4 ± 183.4	0.016	
		*Boswellia* + AvailOm^®^	277.6 ± 148.1	313.2 ± 164.0	0.005	
	Total work for 1RM, J	Placebo	69.9 ± 31.8	70.0 ± 35.5	0.950	0.352
		*Boswellia*	62.2 ± 38.0	65.0 ± 35.7	0.285	
		AvailOm^®^	69.1 ± 37.4	74.9 ± 38.1	0.023	
		*Boswellia* + AvailOm^®^	62.4 ± 31.1	68.0 ± 34.0	0.030	
	Average power, W	Placebo	40.4 ± 22.0	41.8 ± 24.2	0.421	0.050
		*Boswellia*	36.2 ± 24.3	38.1 ± 23.3	0.278	
		AvailOm^®^	40.6 ± 25.3	45.9 ± 25.8	0.003	
		*Boswellia* + AvailOm^®^	35.1 ± 21.3	42.5 ± 25.9	0.001	
At 180°·s^−1^ knee extension	Peak torque, Nm	Placebo	83.3 ± 41.3	82.3 ± 44.2	0.707	0.208
		*Boswellia*	68.1 ± 32.5	72.5 ± 33.8	0.127	
		AvailOm^®^	77.6 ± 33.1	84.9 ± 33.5	0.009	
		*Boswellia* + AvailOm^®^	70.5 ± 29.8	73.8 ± 32.8	0.251	
	Total work, J	Placebo	401.2 ± 200.7	389.7 ± 213.2	0.441	0.050
		*Boswellia*	321.6 ± 174.5	338.5 ± 162.5	0.267	
		AvailOm^®^	372.8 ± 164.9	414.5 ± 172.5	0.005	
		*Boswellia* + AvailOm^®^	342.6 ± 147.1	354.9 ± 159.4	0.413	
	Total work for 1RM, J	Placebo	88.3 ± 42.0	85.1 ± 44.3	0.344	0.050
		*Boswellia*	70.0 ± 38.9	77.2 ± 36.6	0.036	
		AvailOm^®^	81.3 ± 35.4	88.7 ± 34.9	0.024	
		*Boswellia* + AvailOm^®^	76.1 ± 32.4	79.4 ± 34.9	0.316	
	Average power, W	Placebo	133.3 ± 73.8	130.8 ± 80.2	0.641	0.046
		*Boswellia*	106.1 ± 63.4	112.5 ± 58.2	0.248	
		AvailOm^®^	118.4 ± 58.0	137.7 ± 56.9	0.001	
		*Boswellia* + AvailOm^®^	111.9 ± 54.1	119.6 ± 60.8	0.158	
At 180°·s^−1^ knee flexion	Peak torque, Nm	Placebo	51.8 ± 24.4	53.0 ± 25.1	0.491	0.778
		*Boswellia*	46.2 ± 26.6	46.1 ± 24.4	0.988	
		AvailOm^®^	51.9 ± 27.1	54.4 ± 27.1	0.417	
		*Boswellia* + AvailOm^®^	46.9 ± 19.0	48.6 ± 21.4	0.337	
	Total work, J	Placebo	208.2 ± 145.0	203.7 ± 144.9	0.728	0.194
		*Boswellia*	180.9 ± 165.3	182.1 ± 139.7	0.927	
		AvailOm^®^	210.6 ± 151.8	241.7 ± 148.5	0.016	
		*Boswellia* + AvailOm^®^	186.8 ± 124.7	205.5 ± 139.9	0.151	
	Total work for 1RM, J	Placebo	47.5 ± 30.6	46.3 ± 29.1	0.640	0.329
		*Boswellia*	40.9 ± 34.5	42.6 ± 29.3	0.513	
		AvailOm^®^	47.7 ± 30.9	52.1 ± 29.2	0.050	
		*Boswellia* + AvailOm^®^	42.5 ± 25.9	47.2 ± 30.4	0.050	
	Average power, W	Placebo	64.0 ± 49.4	62.2 ± 50.1	0.679	0.096
		*Boswellia*	54.0 ± 49.6	54.9 ± 44.5	0.853	
		AvailOm^®^	62.3 ± 53.0	74.4 ± 48.9	0.008	
		*Boswellia* + AvailOm^®^	54.7 ± 41.7	63.8 ± 49.5	0.046	
**Isometric Dynamometry**						0.725
At 90° knee position	Peak torque, Nm	Placebo	150.3 ± 71.5	153.1 ± 71.1	0.533	
		*Boswellia*	140.7 ± 75.9	146.1 ± 67.1	0.246	
		AvailOm^®^	146.5 ± 73.5	153.3 ± 79.2	0.136	
		*Boswellia* + AvailOm^®^	138.8 ± 66.3	148.9 ± 65.5	0.029	0.620
	Average peak torque, Nm	Placebo	143.2 ± 68.4	143.6 ± 72.6	0.941	
		*Boswellia*	132.2 ± 72.1	138.8 ± 66.8	0.146	
		AvailOm^®^	139.4 ± 72.3	145.7 ± 75.3	0.149	
		*Boswellia* + AvailOm^®^	131.5 ± 63.6	139.6 ± 62.0	0.050	

Data as mean ± standard deviation.

**Table 6:** Results of sleep evaluation by actigraphy in the study groups.

Variable	Baseline	Final (8 Weeks)	Within-Group *p* Value	Between-Group *p* Value
Sleep latency, min				
Placebo	3.3 ± 1.0	3.4 ± 0.8	0.662	0.050
* Boswellia*	3.2 ± 0.9	2.7 ± 1.0	0.016	
AvailOm^®^	3.5 ± 0.8	3.5 ± 0.9	0.843	
* Boswellia* + AvailOm^®^	3.4 ± 1.0	3.0 ± 0.9	0.048	
Sleep efficiency, %				
Placebo	91.5 ± 3.6	91.6 ± 3.2	0.899	0.515
* Boswellia*	90.9 ± 2.7	91.9 ± 2.7	0.050	
AvailOm^®^	91.7 ± 3.6	91.8 ± 3.0	0.755	
* Boswellia* + AvailOm^®^	91.4 ± 3.2	92.2 ± 3.4	0.119	
Total time in bed, min				
Placebo	431.7 ± 60.0	422.1 ± 61.3	0.430	0.462
* Boswellia*	416.2 ± 63.0	427.8 ± 74.0	0.353	
AvailOm^®^	411.0 ± 56.0	405.6 ± 62.2	0.653	
* Boswellia* + AvailOm^®^	419.4 ± 65.0	431.7 ± 72.6	0.318	
Total sleep time, min				
Placebo	396.6 ± 59.6	390.0 ± 61.9	0.563	0.817
* Boswellia*	394.6 ± 68.7	387.2 ± 70.6	0.527	
AvailOm^®^	399.5 ± 82.7	391.1 ± 91.0	0.455	
* Boswellia* + AvailOm^®^	389.2 ± 69.2	394.4 ± 77.6	0.651	
Wakefulness after sleep onset, min				
Placebo	33.3 ± 13.1	33.6 ± 13.1	0.877	0.046
* Boswellia*	37.2 ± 13.0	32.5 ± 10.5	0.019	
AvailOm^®^	30.4 ± 13.3	30.3 ± 11.4	0.947	
* Boswellia* + AvailOm^®^	35.0 ± 10.7	28.9 ± 12.2	0.002	
Number of awakenings				
Placebo	13.8 ± 5.7	13.9 ± 5.5	0.912	0.049
* Boswellia*	16.2 ± 5.0	13.5 ± 5.0	0.009	
AvailOm^®^	15.0 ± 5.8	15.1 ± 5.3	0.954	
* Boswellia* + AvailOm^®^	17.0 ± 6.8	14.1 ± 6.0	0.005	
Awakenings, mean number of min				
Placebo	2.4 ± 0.7	2.4 ± 0.6	0.773	0.750
* Boswellia*	2.3 ± 0.4	2.4 ± 0.5	0.448	
AvailOm^®^	2.5 ± 0.8	2.5 ± 1.0	0.572	
* Boswellia* + AvailOm^®^	2.4 ± 0.7	2.3 ± 0.7	0.591	

Data as mean ± standard deviation; min: minutes.

**Table 7:** Results of the omega-3 index and availability of DHA and EPA in the study groups.

Variable	Baseline	Final (8 Weeks)	Within-Group *p* Value	Between-Group *p* Value
Omega-3 index				
Placebo	5.64 ± 0.88	5.45 ± 0.70	0.251	0.001
* Boswellia*	5.88 ± 1.01	5.50 ± 0.87	0.024	
AvailOm^®^	5.58 ± 1.11	6.10 ± 1.00	0.002	
* Boswellia* + AvailOm^®^	5.35 ± 0.98	6.02 ± 1.42	0.001	
DHA, %				
Placebo	3.29 ± 0.60	3.12 ± 0.49	0.098	0.022
* Boswellia*	3.38 ± 0.60	3.17 ± 0.55	0.038	
AvailOm^®^	3.16 ± 0.70	3.22 ± 0.60	0.507	
* Boswellia* + AvailOm^®^	3.02 ± 0.64	3.19 ± 0.83	0.050	
EPA, %				
Placebo	0.50 ± 0.23	0.51 ± 0.19	0.970	0.001
* Boswellia*	0.63 ± 0.35	0.50 ± 0.28	0.058	
AvailOm^®^	0.59 ± 0.37	0.97 ± 0.39	0.001	
* Boswellia* + AvailOm^®^	0.52 ± 0.27	0.94 ± 0.48	0.001	

DHA: docosahexaenoic acid; EPA: eicosapentaenoic acid.

## Data Availability

Study data are available from the authors upon request.
